# Extraction and analysis of high-quality chloroplast DNA with reduced nuclear DNA for medicinal plants

**DOI:** 10.1186/s12896-024-00843-8

**Published:** 2024-04-18

**Authors:** Yifan Yu, Xinxin Wang, Renjun Qu, Zhen OuYang, Juan Guo, Yujun Zhao, Luqi Huang

**Affiliations:** 1 State Key Laboratory for Quality Ensurance and Sustainable Use of Dao-di Herbs, National Resource Center for Chinese Materia Medica, China Academy of Chinese Medical Sciences, 100700 Beijing, China; 2 School of Pharmacy, Jiangsu University, 212013 Zhenjiang, China

**Keywords:** *Erigeron breviscapus*, Chloroplast isolation, cpDNA extraction, Modified high-salt low-pH method

## Abstract

**Background:**

Obtaining high-quality chloroplast genome sequences requires chloroplast DNA (cpDNA) samples that meet the sequencing requirements. The quality of extracted cpDNA directly impacts the efficiency and accuracy of sequencing analysis. Currently, there are no reported methods for extracting cpDNA from *Erigeron breviscapus*. Therefore, we developed a suitable method for extracting cpDNA from *E. breviscapus* and further verified its applicability to other medicinal plants.

**Results:**

We conducted a comparative analysis of chloroplast isolation and cpDNA extraction using modified high-salt low-pH method, the high-salt method, and the NaOH low-salt method, respectively. Subsequently, the number of cpDNA copies relative to the nuclear DNA (nDNA ) was quantified via qPCR. As anticipated, chloroplasts isolated from *E. breviscapus* using the modified high-salt low-pH method exhibited intact structures with minimal cell debris. Moreover, the concentration, purity, and quality of *E. breviscapus* cpDNA extracted through this method surpassed those obtained from the other two methods. Furthermore, qPCR analysis confirmed that the modified high-salt low-pH method effectively minimized nDNA contamination in the extracted cpDNA. We then applied the developed modified high-salt low-pH method to other medicinal plant species, including *Mentha haplocalyx*, *Taraxacum mongolicum*, and *Portulaca oleracea*. The resultant effect on chloroplast isolation and cpDNA extraction further validated the generalizability and efficacy of this method across different plant species.

**Conclusions:**

The modified high-salt low-pH method represents a reliable approach for obtaining high-quality cpDNA from *E. breviscapus.* Its universal applicability establishes a solid foundation for chloroplast genome sequencing and analysis of this species. Moreover, it serves as a benchmark for developing similar methods to extract chloroplast genomes from other medicinal plants.

**Supplementary Information:**

The online version contains supplementary material available at 10.1186/s12896-024-00843-8.

## Background

Chloroplasts serve as the primary organelles for photosynthesis in plants. As a semi-autonomous genetic system, they harbor relatively independent genetic material known as chloroplast DNA (cpDNA). Widely utilized for plant genetic enhancement and phylogenetic studies, cpDNA plays a pivotal role in these fields. The plant chloroplast genome typically consists of a circular molecule approximately 150 kb in size, containing 120–130 genes ranging from 72 to 217 kb in size [1, 2]. Remarkably conserved in content and structure, the plant chloroplast genome furnishes ample information for species evolutionary analysis and genetic improvement endeavors [2–7]. In recent years, the rapid advancement of high-throughput sequencing technology has led to significant strides in sequencing plant chloroplast genomes. However, the acquisition of high-quality chloroplast genome data hinges not only on sequencing technology but also on the availability of cpDNA samples meeting stringent sequencing requirements. High-quality cpDNA is extracted from intact chloroplasts with minimal nuclear and organelle genomes contamination. Such cpDNA facilitates the generation of clean reads, thereby reducing sequencing challenges and aiding subsequent analyses, particularly de novo assembly of short reads [8, 9]. Consequently, the development of efficient and species-specific cpDNA extraction methods is imperative for successful sequencing endeavors [10, 11].

Traditional methods for plant cpDNA extraction include PCR (polymerase chain reaction) [4, 12–14], sucrose density gradient centrifugation [15, 16], percoll density gradient centrifugation [17], and high-salt methods [18]. PCR is generally suitable for extracting total DNA from a limited plant material, requiring the use of conserved primer pairs [19] to amplify the chloroplast genome further. However, this process is time-consuming and challenging due to genetic organization differences among plant species [20]. Moreover, the presence of “promiscuous” DNA transferred from chloroplasts and mitochondria to the nucleus can affects the reliability [21–24].

Traditional density gradient centrifugation methods are more suitable for extracting chloroplast genomes from grasses and legumes. However, they are time-consuming, labor-intensive, and often yield the low DNA quantities [25], limiting their widespread use. While some improved cpDNA extraction methods like high-salt low-pH [26] and NaOH low-salt methods [27] have emerged in recent years, showing promise in yielding intact chloroplasts and high-quality cpDNA quickly and easily, there is still no universal method applicable to all plant species. Therefore, there is an urgent need to develop species-specific protocols to enhance cpDNA quality and yield while simplifying operational procedures [28].

*E. breviscapus*, a renowned medicinal plant in the Asteraceae family, holds significant economic and medicinal value, particularly in southwestern China [29]. However, rampant overexploitation and misuse have led to sever depletion of its wild resources. Hence, genetic engineering improvement and genetic diversity analysis serve as effective strategies for conserving and scientifically harnessing the germplasm resources of *E. breviscapus*.

Currently, no methods have been reported for the extraction cpDNA from *E. breviscapus*. Therefore, obtaining high-quality *E. breviscapus* cpDNA through appropriate methods is crucial for the conserving and scientifically utilizing its germplasm resources. To address this gap, our study established an efficient method for chloroplast isolation and cpDNA extraction of *E. breviscapus*. Furthermore, to assess the its generalizability, we tested its effectiveness on several common medicinal plants. The genus *Mint* and *Taraxacum* pose challenges in phylogenetic studies due to their complex hybridity and coexistence of agamosperms [30–32]. Sequencing high-quality chloroplast genome sequences of *M. haplocalyx* and *T. mongolicum* would provide valuable genetic resource for molecular studies and phylogenetic analysis within the Labiatae and Asteraceae families, respectively.

*P. oleracea*, recognized by the WHO as one of the most utilized medicinal and edible plants, stands to benefit from acquiring a high-quality chloroplast genome. Such data would enhance understanding of active ingredient in Traditional Chinese Medicine (TCM) and facilitate drug development for this species [30, 31]. Additionally, the leathery and thicker leaves of *P. oleracea* make it suitable for testing the generalizability of our method.

This study lays the foundation for direct sequencing of the chloroplast genome of *E. breviscapus*, facilitating further evolutionary analysis and genetic improvement of this species. Moreover, it provides a reference for chloroplast isolation and cpDNA extraction in other medicinal plant species.

## Results

### Isolation of chloroplasts from leaf tissue

**Fig. 1:**
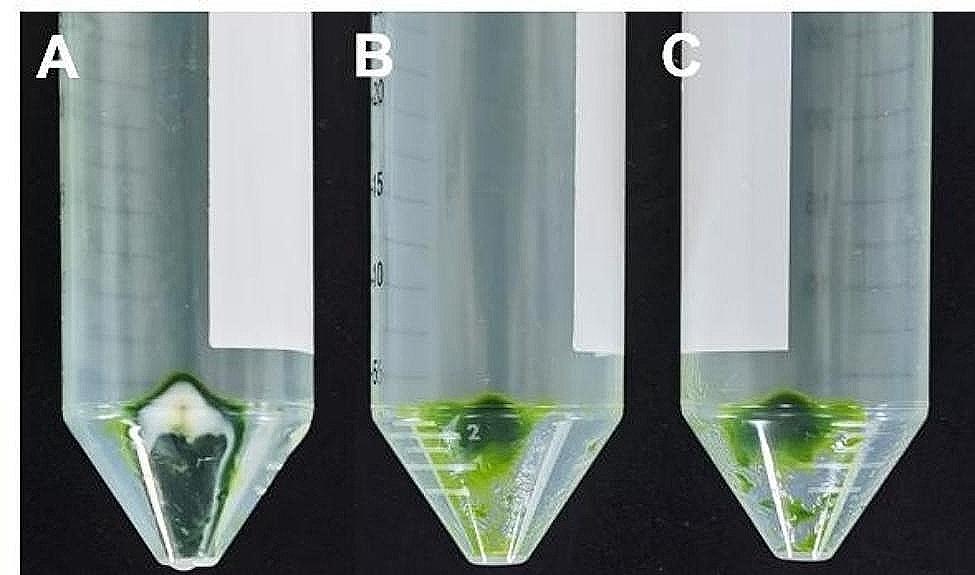
Comparison of chloroplast precipitates isolated by different chloroplast isolation methods. Chloroplast precipitates isolated by the high-salt method (**A**). Chloroplast precipitates isolated by the modified high-salt low-pH method (**B**). Chloroplast precipitates isolated by the NaOH low-salt method (**C**)

Chloroplast precipitates were isolated using different centrifugation strategies, and the yield and purity of isolated chloroplasts from *E. breviscapus* were visually analyzed. As illustrated in Fig. 1, the significant numbers of chloroplasts could be obtained through the high-salt method, the modified high-salt low-pH method, and the NaOH low-salt method. While the high-salt method yielded a higher number of chloroplasts compared to the modified high-salt low-pH method and the NaOH low-salt method, the chloroplast precipitates obtained via the high-salt method contained a considerable amount of cell debris and other impurities (Fig. 1A). In contrast, chloroplast precipitates obtained by the other two methods were noticeably purer, with fewer impurities present (Fig. 1B and C).

### Microscopic observation of chloroplasts

**Fig. 2:**
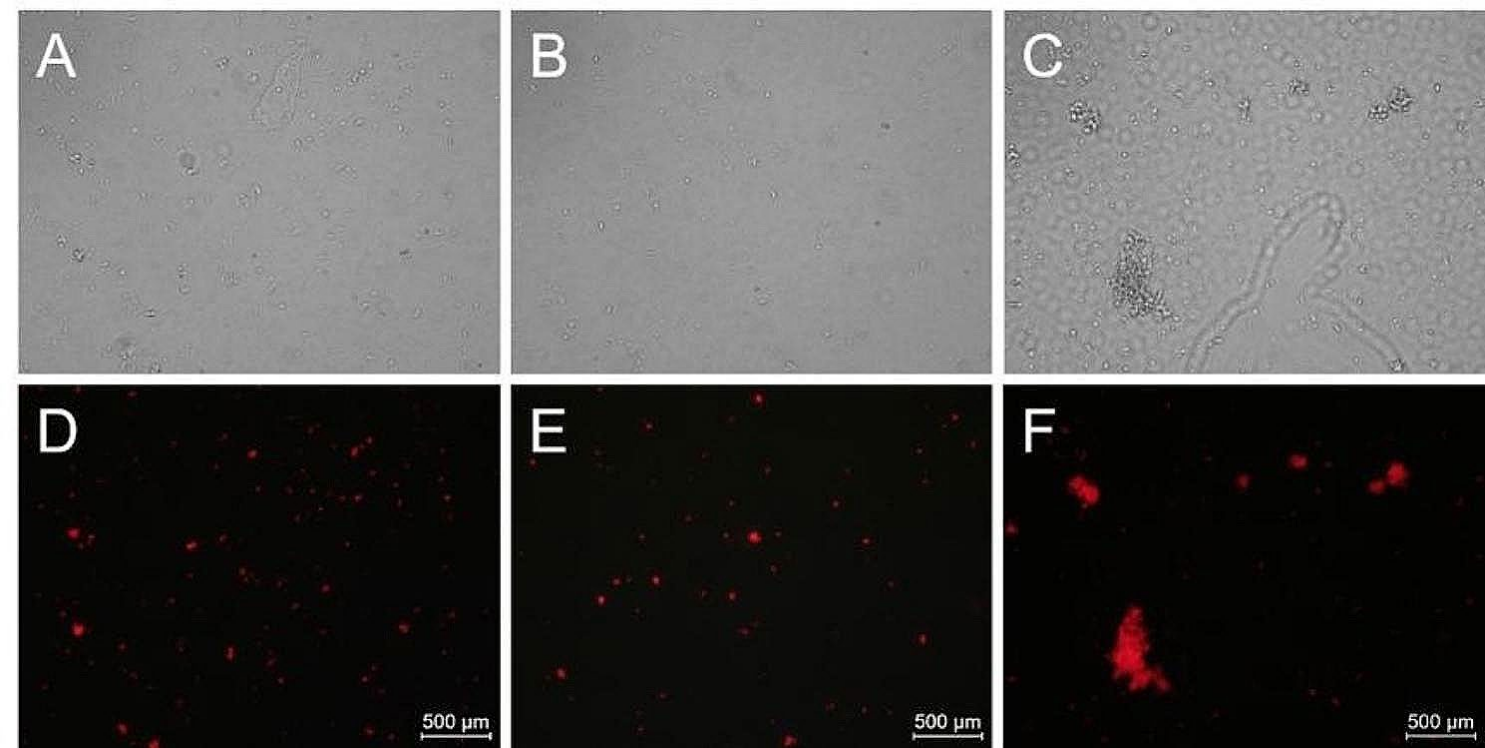
Brightfield and fluorescence microscopy images of isolated chloroplasts. Chloroplasts isolated using the modified high-salt low-pH method (**A**, **D**). Chloroplasts isolated using the NaOH low-salt method (**B**, **E**). Chloroplasts isolated using the high-salt method (**C, F**). Scale bars = 500 μm

The yield and integrity of isolated chloroplasts from *E. breviscapus* were visually examined under an inverted fluorescence microscope (Fig. 2). Although a significant number of chloroplasts were visible in the natural light field (Fig. 2C), chlorophyll fluorescence (red) was not observed in most of the corresponding locations in the dark field (Fig. 2F). Instead, chlorophyll fluorescence was only visible in a few locations, indicating that most chloroplasts isolated by the high-salt method were damaged. Furthermore, numerous areas of chloroplast aggregation and non-chloroplast organelles were visible in both the natural light field (Fig. 2C) and dark field (Fig. 2F), suggesting that the high-salt method failed to effectively remove excess cellular fractions.

In contrast, chloroplasts isolated by the modified high-salt low-pH and NaOH low-salt methods appeared uniformly dispersed with clear backgrounds in the majority of the natural light field (Fig. 2A and B). The distribution of chloroplasts in the natural light field closed matched the location of chlorophyll fluorescence in the dark field (Fig. 2D and E), indicating that both methods successfully isolated intact chloroplasts.

.

### Concentration and quality determination of cpDNA

For purified DNA, the A_260 nm_/A_280 nm_ ratio typically falls between 1.8 and 2.0, serving as a gauge of DNA purity. A ratio below 1.8 suggests the presence of protein or other contaminants, while a ratio above 2.0 indicates the presence of RNA or other organic contaminants. Moreover, if the A_260 nm_/A_230 nm_ value for a DNA sample is below 2.0, it signifies contaminated with carbohydrates, salts, or organic solvents. Thus, the optimal values for high-quality DNA range from 1.8 to 2.0.

For cpDNA extracted using the modified high-salt low-pH method, the A_260nm_/A_280nm_ ratio was 1.83, indicating the absence of protein, phenol, and polysaccharide contamination. The A_260nm_/A_230nm_ ratio was 1.96, suggesting low levels of carbohydrates, salts, or organic solvents in the cpDNA sample, with a concentration of 1598.6 ng/µL. In contrast, for cpDNA extracted using the high-salt method, the A_260nm_/A_280nm_ ratio was 2.09, suggesting the presence of RNA or other organic contaminants. The A_260nm_/A_230nm_ ratio was 0.83, indicating high levels of carbohydrates, salts, or organic solvents, with a concentration of the cpDNA sample was 563.9 ng/µL. Similarly, for cpDNA extracted using the NaOH low-salt method, the A_260nm_/A_280nm_ ratio was 1.99, indicating the potential presence of RNA or other organic contaminants. The A_260nm_/A_230nm_ ratio was 0.92, indicating contamination with carbohydrates, salts, or organic solvents, with a concentration of 241.0 ng/µL. Notably, the high-salt method and NaOH low-salt method filed to meet the target value of 1.8 for A_260nm_/A_280nm_ and 2.0 for A_260nm_/A_230nm_, respectively. Moreover, the concentration of cpDNA extracted by these two methods was lower than that obtained using the modified high-salt low-pH method (Table 1).

**Table 1:** Median DNA yield (ng/µL) and quality (A_260nm_/A_280nm_ and A_260nm_/A_230nm_) of three extraction methods tested on six individual *E. breviscapus* samples

Method	Median (Range) DNA concentration (ng/µL)	Median (Range) DNA Quality A_260nm_/A_280nm_	Median (Range) DNA Quality A_260nm_/A_230nm_
Modified high-salt low-pH method	1598.6 (1054.2–1659.0)	1.83 (1.80–1.88)	1.96 (1.91–2.01)
NaOH low-salt method	241.0 (229.7-261.5)	1.99 (1.97-2.00)	0.92 (0.87–0.99)
High-salt method	563.9 (540.4-589.5)	2.09 (2.07–2.11)	0.83 (0.81–0.94)

### Detection of cpDNA by agarose gel electrophoresis

**Fig. 3:**
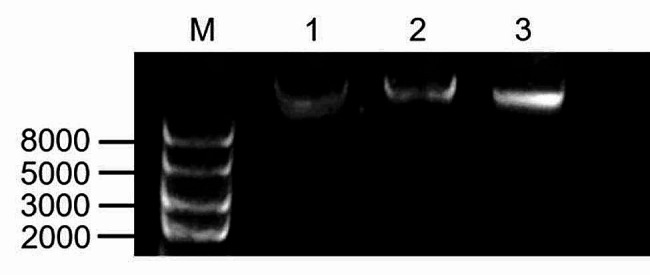
Electrophoresis pattern of cpDNA extracted by different chloroplast isolation methods. Trans2K® Plus II DNA marker (**M**) is shown as reference. Electrophoresis pattern of cpDNA extracted by the high-salt method (**1**). Electrophoresis pattern of cpDNA extracted by the NaOH low-salt method (**2**). Electrophoresis pattern of cpDNA extracted by the modified high-salt low-pH method (**3**)

We further assessed the quality of cpDNA extracted by the modified high-salt low-pH method, the NaOH low-salt method, and the high-salt method using agarose gel electrophoresis. As shown in Fig. 3, the cpDNA sample extracted by the modified high-salt low-pH method appeared clear and bright, with no evident tailing. In contrast, the brightness of the cpDNA samples extracted by the NaOH low-salt method and the high-salt method was comparatively lower, with the cpDNA sample extracted by the high-salt method showing signs of degradation. Furthermore, a faint light background smear was visible at the bottom of the lane containing the cpDNA sample extracted by the high-salt method. These results indicated that the cpDNA sample obtained by the modified high-salt low-pH method exhibited good integrity and minimal degradation. Conversely, the quality of cpDNA extracted by the high-salt method and NaOH low-salt method was inferior to that of the modified high-salt low-pH method. Notably, the high-salt method appeared to induce degradation of the extracted cpDNA.

### Calculation of chloroplast and nDNA by qPCR

**Fig. 4:**
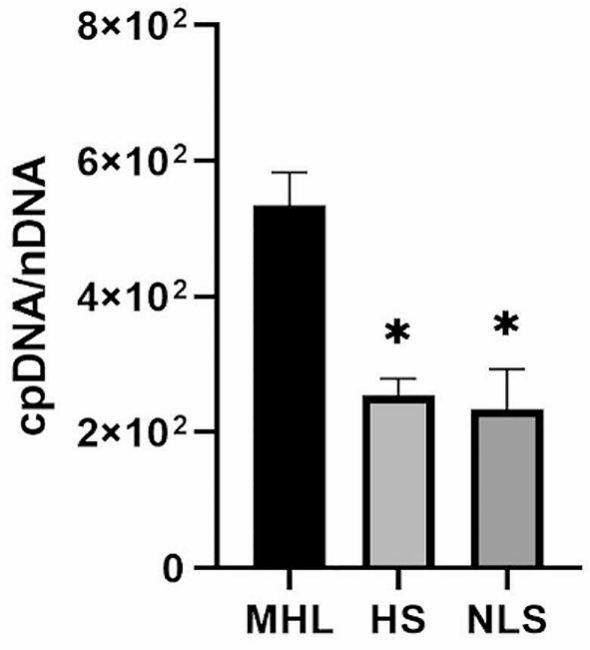
Comparison of the purity of extracted cpDNA using different chloroplast isolation methods. Modified high-salt low-pH method **(MHL)**; High-salt method **(HS)**; NaOH low-salt method **(NLS).** Data are expressed as mean ± SE (*n* = 3). The asterisk indicates a significant difference (*p < 0.05)

qPCR was employed to determine the copy number of cpDNA relative to the nDNA, as illustrated in Fig. 4. The copy number of genes amplified from each genome (chloroplast and nuclear) was quantified. Based on these values, the contamination percentage of cpDNA with nDNA copies was calculated. The results revealed that the purest cpDNA preparation was obtained from chloroplast extracts using the modified high-salt low pH method, exhibiting a significant reduction (*p* < 0.05) in both nDNA contaminations compared to other methods. Specifically, the modified high-salt low-pH method reduced nuclear contamination to approximately 52.4% and 58.3% compared to the high-salt and NaOH low-salt methods, respectively (Fig. 4).

### Application of the modified high-salt low-pH method

**Fig. 5:**
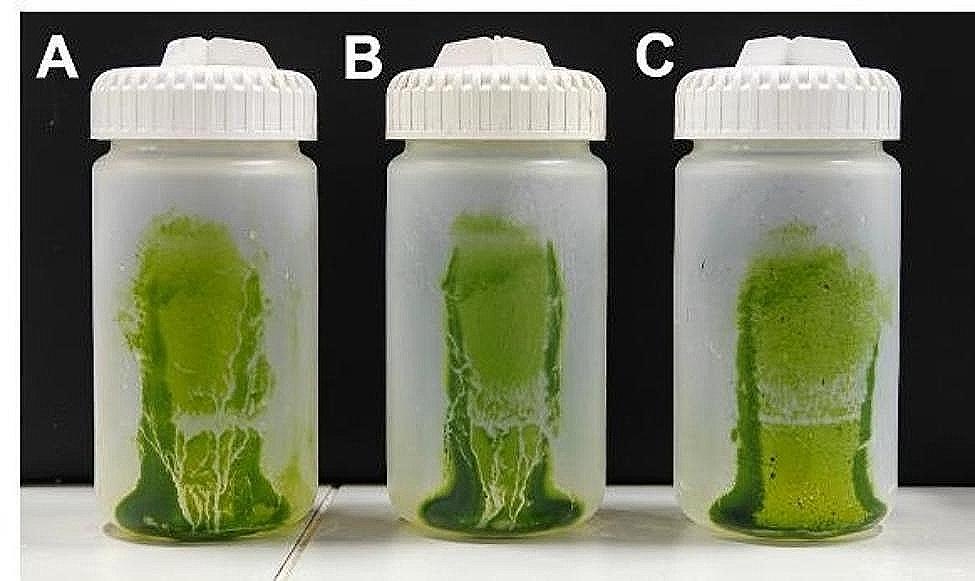
Comparison of isolated chloroplast precipitates using the modified high-salt low-pH method from different species. Isolated chloroplast precipitates from *M. haplocalyx* using the modified high-salt low-pH method (**A**). Isolated chloroplast precipitates from *P. oleracea* using the modified high-salt low-pH method (**B**). Isolated chloroplast precipitates from *T. mongolicum* using the modified high-salt low-pH method (**C**)

**Fig. 6:**
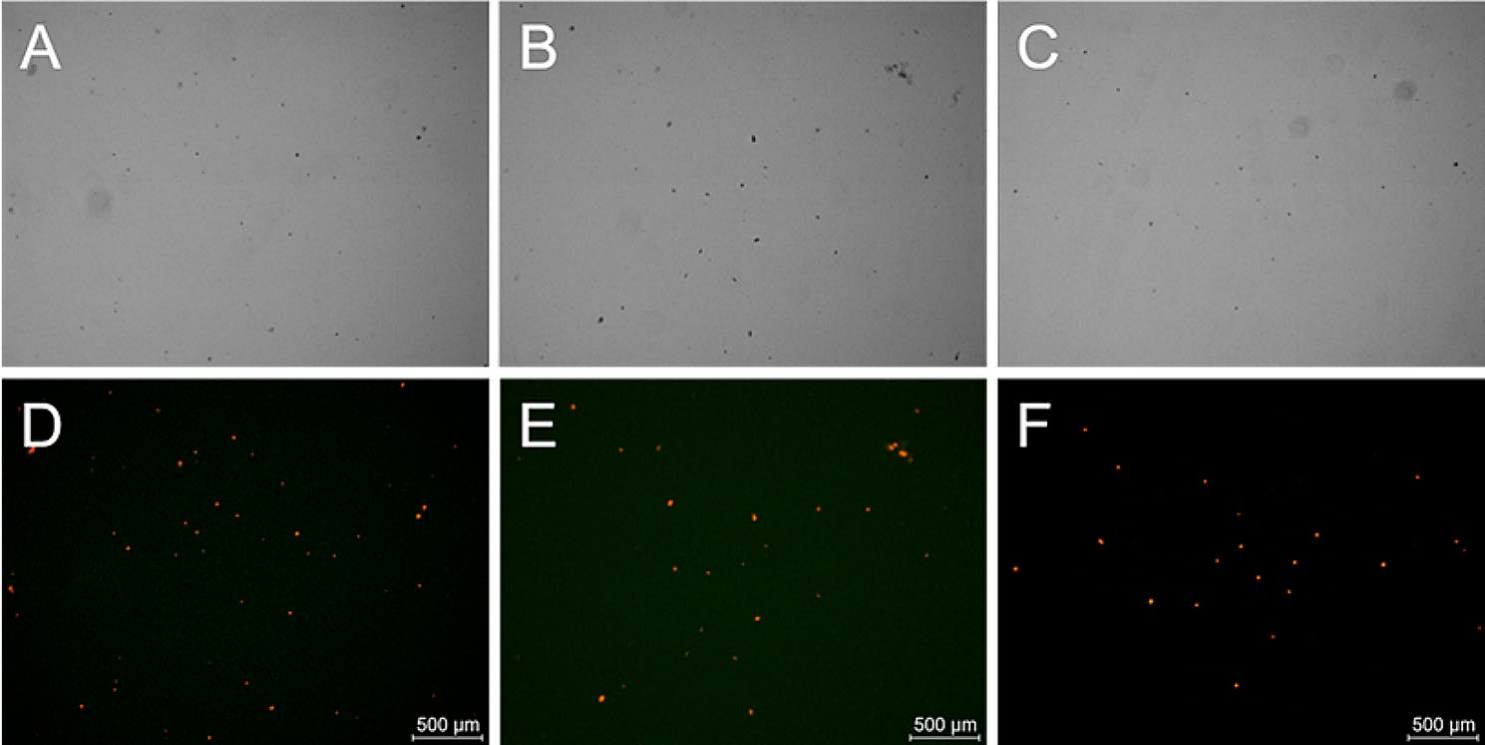
Brightfield and fluorescence microscopy images of isolated chloroplasts from different species using the modified high-salt low-pH method. Chloroplasts isolated from *M. haplocalyx* (**A, D**). Chloroplasts isolated from *P. oleracea* (**B, E**). Chloroplasts isolated from *T. mongolicum* (**C, F**). Scale bars = 500 μm

To further validate the generalizability of the modified high-salt low-pH method, we applied it to isolate chloroplasts and extract cpDNA from *M. haplocalyx*, *P. oleracea*, and *T. mongolicum*. As shown in Fig. 5, the modified high-salt low-pH method yielded a substantial number of chloroplasts from these species with fewer impurities and higher purity. Under natural light, chloroplasts from *M. haplocalyx*, *P. oleracea*, and *T. mongolicum* were uniformly distributed with a clear background and devoid of contamination by other non-chloroplast organelles and cellular debris (Fig. 6A, B and C). This indicates that the method is equally effective in isolating chloroplasts from the leaves of these species and obtaining chloroplast precipitates of high purity. Moreover, the positions of chloroplasts isolated from *M. haplocalyx* (Fig. 6A), *P. oleracea* (Fig. 6B), and *T. mongolicum* (Fig. 6C) in the bright light field using the modified high-salt low-pH method closely matched the positions of chloroplast autofluorescence in the dark field (Fig. 6D, E and F). This suggests the method’s applicability in isolating structurally intact chloroplasts from medicinal plants.

High concentrations of cpDNA were extracted from *E. breviscapus*, *M. haplocalyx*, *P. oleracea*, and *T. mongolicum* using the modified high-salt low-pH methods, with values ranging from 1598.6 to 2612.8 ng/µL. All modified high-salt low-pH methods produced cpDNA with A_260_ nm/A_280_ nm value ranging from 1.83 to 1.98 (Table 2), with *E. breviscapus* cpDNA consistently closest to the optimal value (1.83). However, there were no significant differences in A_260 nm_/A_280 nm_ measurements between cpDNA from different species. Similarly, all modified high-salt low-pH methods produced cpDNA with A_260 nm_/A_230 nm_ value between 1.96 and 2.20 (Table 2), with *E. breviscapus* cpDNA consistently around the optimal value (1.96). Again, there were no significant differences in A_260 nm_/A_230 nm_ measurements between cpDNA from different species, indicating the effectiveness of the modified high-salt low-pH method in extracting high-quality and high-purity cpDNA samples from various medicinal plants, demonstrating its generalizability.

**Table 2:** Median DNA yield (ng/µL) and quality (A_260nm_/A_280nm_ and A_260nm_/A_230nm_) of the modified high-salt low-pH method tested on six individual samples from different species

Species	Median (Range) DNA concentration (ng/µL)	Median (Range) DNA Quality A_260nm_/A_280nm_	Median (Range) DNA Quality A_260nm_/A_230nm_
*Erigeron breviscapus*	1598.6 (1054.2–1659.0)	1.83 (1.80–1.88)	1.96 (1.91–2.01)
*Mentha haplocalyx*	2145.6 (1943.7-2276.3)	1.97 (1.93–1.98)	2.13 (2.11–2.14)
*Portulaca oleracea*	2612.8 (2428.0-2804.7)	1.98 (1.96–1.99)	2.20 (2.19–2.21)
*Taraxacum mongolicum*	1697.6 (1532.4-1874.5)	1.90 (1.89–1.93)	2.02 (2.01–2.04)

The copy number of cpDNA relative to the nDNA was calculated by qPCR for the other three species. The (cpE(cpCt))/(nE(nCt)) value of the cpDNA sample from *E. breviscapus* was 535.1, from *M. haplocalyx* was 234.4, from *P. oleracea* was 157.3, from *T. mongolicum* was 194.4. The copy number of cpDNA from *E. breviscapus*, *M. haplocalyx*, *P. oleracea*, and *T. mongolicum* were all higher relative to the nDNA copy number using this method, indicating the generalizability of this approach.

## Discussion

The process of obtaining high-quality cpDNA in plants involved three crucial steps: isolation of intact chloroplasts from leaf tissue, sufficient lysis of the chloroplasts, and purification of the cpDNA. Among these steps, the isolation of intact chloroplasts is typically the most critical for obtaining high-quality cpDNA.

During the homogenization process, the high salt ion environment provided by buffer A effectively reduces the electrostatic effects generated by homogenization. This reduction prevents the adsorption of undissolved nDNA and chromatin to the chloroplast outer membrane, thereby minimizing nDNA contamination. Conversely, the NaOH low-salt method reduced the salt content, potentially leading to nDNA contamination in the extracted cpDNA. Figure 4 illustrates that the modified high-salt low-pH method exhibits the least nDNA contamination, while the NaOH low-salt method demonstrates the most severe contamination. Moreover, the acidic isolation solution with pH 3.8 effectively prevents the ionization and oxidation of polyphenols to quinones, which could otherwise cause DNA degradation. While the NaOH low-salt method may provide a chloroplast isolation effect similar to that of the modified high-salt low-pH method (Figs. 1B and C and 2A and B), prolonged alkaline pretreatment may oxidize phenols to quinones, resulting in lower cpDNA extraction yields compared to the modified high-salt low-pH method (Table 1).

In previous studies [26], it was observed that centrifugation at 200*g* twice was insufficient to completely precipitate the nuclei and cell wall debris, resulting in reduced the chloroplasts recovery rate. Therefore, we adjusted the centrifugal force parameters accordingly. The homogenate was centrifuged once at low speed (500*g* for 20 min) to pellet the nuclei and cell wall debris, followed by centrifuged of the supernatant at high speed (2,500*g* for 12 min) to completely pellet the chloroplasts. This adjustment further reduced the contamination by nDNA attached to the outer membrane of the chloroplast. Consequently, the nDNA contamination of the modified high-salt low-pH method was significantly lower than that of the high-salt method at the same NaCl concentration (Fig. 4).

Buffer B served as the suspension buffer for washing the chloroplast pellet and further enhancing the purity of the cpDNA. PVP-40 in buffer B was acted as a specific adsorbent for phenolic substances. Additionally, the inclusion of reducing agents such as ascorbic acid, sodium metabisulfite, BSA, and DTT not only effectively prevented the oxidation of phenolic substances but also eliminated the strong, irritating odor and toxicity associated with of β-mercaptoethanol in the high-salt method and NaOH low-salt method. Moreover, these reagents helped protect the chloroplast membrane structure to some extent.

Microscopy images (Fig. 2A, D, B and E) from the modified high-salt low-pH and NaOH low-salt methods demonstrated that after the isolation and purification steps, numerous intact chloroplasts were observed with only a few fragmented chloroplasts visible in the natural light field. The background was clear, and there was no evidence of contamination. In contrast, the high-salt method lacked effective isolation and purification steps, leading to a significant presence of fragmented chloroplasts and cell debris in the microscopic images (Fig. 2C and F).

Finally, we opted to used SDS and proteinase K instead of CTAB to lyse the purified chloroplasts. SDS was an anionic surfactant, could effectively lyse cells at higher temperatures (55 °C), isolate chromosomes, and denature proteins. Furthermore, SDS could bind to proteins and polysaccharides, facilitating the release nucleic acids. By increasing the salt concentration and lowering the temperature, the solubility of the SDS-protein complex would decrease, leading to the complete precipitation of protein and polysaccharide contaminants. Proteinase K, a serine protease with broad cleavage active in a pH range from 4.0 to pH 12.0, remained stable in presence of SDS and EDTA. Thus, nuclease could be removed during the chloroplast cleavage process, effectively preventing the degradation of cpDNA. The concentration determination and quality control demonstrated higher cpDNA yields for the modified high-salt low-pH method. The method consistently yields ideal A_260 nm_/A_280 nm_ and A_260 nm_/A_230 nm_ ratios of 1.83 and 1.96, respectively, indicating good quality and high purity of the cpDNA extracted, meeting the the standard for subsequent chloroplast genome sequencing. However, contamination with polysaccharides and polyphenols was observed in the other two evaluated methods, indicating that the high-salt method and NaOH low-salt methods were not suitable for obtaining high-quality cpDNA from *E. breviscapus* (Table 1).

Gel electrophoresis results (Fig. 3) further confirmed the integrity of the cpDNA extracted by the modified high-salt low-pH method. The cpDNA sample obtained by this method exhibited clarity and brightness significantly superior to that of the NaOH low-salt method, with no obvious tailing. These observations indicated that the cpDNA obtained by the modified high-salt low-pH method possessed good integrity and showed no signs of degradation. Overall, both the quality and yield of the cpDNA extracted by this method surpassed those obtained by the high-salt method and the NaOH low-salt method.

Using the modified high-salt low-pH method to isolate chloroplasts and extract cpDNA from *E. breviscapus* and other medicinal plants, we found that the method effectively isolated chloroplasts and maintained chloroplast integrity, yielding ideal cpDNA samples from all species (Table 2). However, the level of nDNA contamination in the cpDNA samples obtained varied, likely due to the differences in leaf morphology and physicochemical properties among *E. breviscapus*, *M. haplocalyx*, *P. oleracea*, and *T. mongolicum*. *E. breviscapus* leaves were the softest and thinnest, while those of *M. haplocalyx* and *T. mongolicum* were slightly tougher and thicker, and those of *P. oleracea* were notably tougher and thicker than the others. Consequently, using the same homogenization time might result in varying homogenization effects. *E. breviscapus* exhibited the highest copy number of cpDNA relative to nDNA, followed by *M. haplocalyx* and *T. mongolicum*, while the copy number of *P. oleracea* cpDNA relative to nDNA was significantly lower than that of the other three species, consistent with our hypothesis.

## Conclusions

In this study, we appropriately modified the high-salt low-pH method and thoroughly compared it with the high-salt method and the NaOH low-pH method in terms of chloroplast isolation efficacy and cpDNA extraction efficiency. Our investigation aimed to determine the generalizability of the modified high-salt low-pH method. The results demonstrated that the modified high-salt low-pH method is indeed generalizable, effectively isolating chloroplasts from mature leaves of *E. breviscapus*, *M. haplocalyx*, *P. oleracea*, and *T. mongolicum* while preserving the integrity of chloroplast structure.

The yield, quality, and purity of cpDNA obtained using the modified high-salt low-pH method were significantly superior to those obtained using the high-salt method and the NaOH low-salt method. Consequently, we have successfully developed a versatile method suitable for extracting high-quality cpDNA from mature leaves of medicinal plants. Moreover, we have provided detailed discussions on the operating procedures, parameter settings, and the rationale behind reagent selection, offering valuable insights for future research on cpDNA extraction from other medicinal plants.

## Materials and methods

### Plant materials

The seeds of *Erigeron breviscapus* were stored at the State Key Laboratory for Quality Ensurance and Sustainable Use of Dao-di Herbs in Beijing, China. Before inoculation, the seeds were soaked in deionized water for 3–4 h and dried at room temperature for another 3–4 h. Subsequently, they were then soaked in 1% v/v NaClO solution (Solarbio, China) for 10 min, followed by three rinses with sterile water under sterile conditions. Approximately 20–30 sterilized seeds per Petri dish were then inoculated into MS (Murashige and Skoog) medium (PhytoTech Labs, M519, USA) [32] for germination. The germinated seeds were cultured in a growth chamber under 30 µmol photons/m^2^/s, with a 16 h light/8 h dark cycle at 25 °C for 4–7 days.

Under germination, the seedlings are transferred to sterile tissue culture bottles containing MS medium supplemented with 30 g/L sucrose (Sinopharm Chemical Reagent Co., Ltd., China) and maintained in a growth chamber under 30 µmol photons/m^2^/s, with a 16 h light/8 h dark cycle at 26 °C for 4–8 weeks [33]. The mature plants of *Erigeron breviscapus* (Vant.) Hand-Mazz., *Mentha haplocalyx* Briq., *Taraxacum mongolicum* Hand-Mazz and *Portulaca oleracea* Linn. were obtained from the phytotron of the State Key Laboratory for Quality Ensurance and Sustainable Use of Dao-di Herbs on April 2, 2023. To ensure successful isolation of intact plastids during cpDNA extraction, the mature plants of *E. breviscapus*, *M. haplocalyx*, *P. oleracea*, and *T. mongolicum* were subjected to a dark period of 48–72 h at 4 °C. This step was implemented to reduce starch accumulation, as starch has been shown to interfere with the subsequent process of chloroplast isolation [34, 35].

### Isolation of chloroplast

The chloroplasts of *E. breviscapus* were isolated using the high-salt method [18], the NaOH low-salt method [27], and the modified high-salt low-pH method. For *M. haplocalyx*, *P. oleracea*, and *T. mongolicum*, chloroplast isolation was performed exclusively with the modified high-salt low-pH method. The components of all the buffers necessary for these methods were procured from SCRC and are detailed in Table 3.

**Table 3:** Reagents utilized in the modified high-salt low-pH method, high-salt method, and NaOH low-salt method

Method	Buffer	Components
Modified high-salt low-pH method	Buffer A	1.25 M NaCl, 0.25 M Ascorbic acid, 10 mM Sodium metabisulfite, 50 mM Tris [tris (hydroxymethyl) aminomethane hydrochloride] -HCl (pH 8.0), 7 mM EDTA (ethylene diamine tetraacetic acid), 1% w/v PVP (polyvinylpyrrolidone)-40, 0.1% w/v BSA (bovine serum albumin), 1 mM DTT (dithiothreitol)
	Buffer B	1.25 M NaCl, 50 mM Tris-HCl (pH 8.0), 25 mM EDTA (pH 8.0), 1% w/v PVP-40, 0.1% w/v BSA, 1 mM DTT
	Buffer C	50 mM Tris-HCl, 25 mM EDTA (pH 8.0), 1.25 M NaCl, 2.0% SDS (sodium dodecyl sulfate)
High-salt method	Buffer D	1.25 M NaCl, 50 mM Tris-HCl (pH 8.0), 5 mM EDTA, 0.1% w/v BSA, 0.1% v/v β-mercaptoethanol
NaOH low-salt method	Buffer E	0.5 M NaOH, 7 mM EDTA, 1 g PVP-40, 0.2% v/v β-mercaptoethanol
	Buffer F	0.62 M NaCl, 50 mM Tris-HCl, 7 mM EDTA, 2 g PVP-40, 1.5 mM DTT
	Buffer G	0.35 M Sorbitol, 50 mM Tris-HCl, 25 mM EDTA

**Fig. 7:**
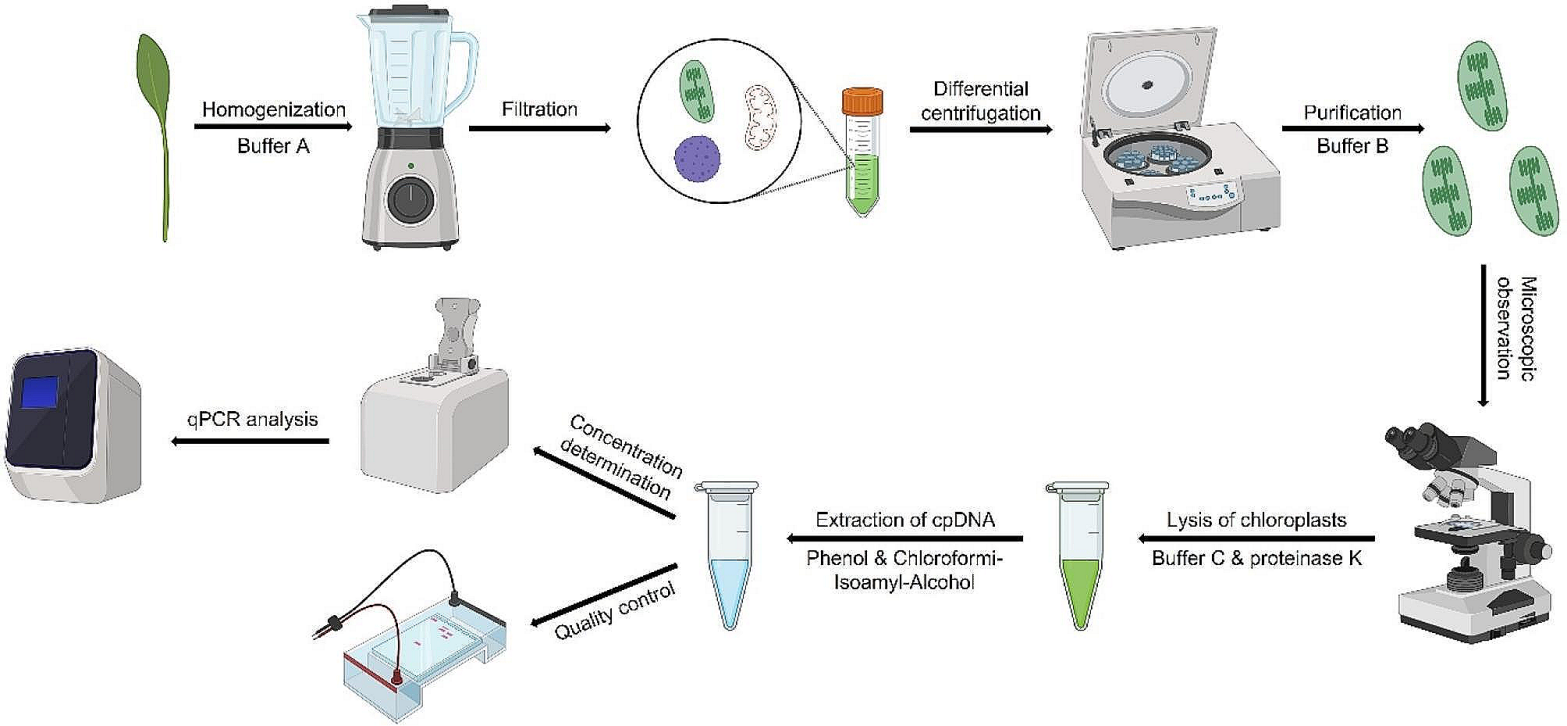
Flowchart detailing the essential steps in chloroplast DNA (cpDNA) isolation and analysis

All centrifugation steps were carried out at 4 °C unless otherwise specified. In the modified high-salt low-pH method (Fig. 7), the primary leaf veins of the pre-treated leaves were excised, and the leaves were cut into approximately 1 cm pieces. For every 20 g of leaves, 100 mL of pre-cooled buffer A was added, and the mixture was thoroughly homogenized using a homogenizer (Midea, China). The resulting homogenate was filtered twice through four layers of gauze, and the filtrate was then evenly distributed into 50 mL centrifuge tubes, with 25 mL in each tube. Chloroplasts were isolated through differential centrifugation (500*g*, 20 min). The supernatant was carefully transferred to fresh centrifuge tube and centrifuged at 2,500*g* for 12 min. Subsequently, the supernatant was decanted, and the green pellet (the resulting chloroplast precipitate) was retained. It is important to protect the chloroplast precipitate from light to prevent starch aggregation. To purify the chloroplast pellet, 25 mL of pre-cooled buffer B was added, gently mixed using a sterile soft brush, and then centrifuged at 2,500*g* for 12 min. The supernatant was discarded, and this step was repeated once more. The resulting precipitate represented the purified chloroplast, which were then stored at 4 °C.

For the high-salt method, 20 g of leaves were sliced into 1 cm pieces and homogenized with 100 mL of pre-cooled buffer D for 30 s. The resulting homogenate was filtered through two layers of gauze, and then the filtrate was centrifuged at 3,000*g* for 10 min. The chloroplast pellet obtained was resuspended in 30 mL of pre-cooled buffer D for 30 s and centrifuged again at 3,000*g* for 10 min. Finally, the chloroplast pellet was resuspended in 10 mL of pre-cooled buffer D for 30 s and stored at 4 °C.

For the NaOH low-salt method, 20 g of leaves underwent pretreatment with 300 mL buffer E and shaken at 37 °C for 40 min. Subsequently, the pretreated leaves were thoroughly washed with deionized water until the pH reached 7.0. The leaves were then homogenized with 200 mL of pre-cooled buffer F. The resulting homogenate was filtered twice through two layers of gauze and centrifuged at 200*g* for 20 min to remove cell debris. The supernatant was further centrifuged at 3,000*g* for 20 min to precipitate the released chloroplasts. The chloroplast pellets obtained were resuspended in 200 mL of buffer G, centrifuged again at 3,000*g* for 20 min, and finally, the chloroplast pellets were resuspended in 10 mL of buffer G and stored at 4 °C.

### Microscopic observation of chloroplasts

Microscopic examination of chloroplasts was conducted to assess their integrity and detect chlorophyll fluorescence using an inverted fluorescence microscope (Zeiss, Axio Vert.A1, Germany). In brief, 10 µL of purified chloroplast suspensions were placed on temporary glass slides and observed under the microscope to assess chloroplast morphology under natural light. The excitation light wavelength was set to 470 nm, and the chlorophyll fluorescence emission was detected within the range of 650–682 nm. Chlorophyll fluorescence (appearing as red) was observed in the dark field, and image from both the natural light and the dark field were merged using ZEN software 3.7 (Zeiss, Germany).

### Extraction of cpDNA

To the purified chloroplasts obtained using the modified high-salt method, 10 mL buffer C was added. Subsequently, 30–40 µL proteinase K (20 mg/mL) (Solarbio, China) was added and the mixture was vigorously shaken. It was then incubated at 55 °C for 3–5 h to ensure sufficient lysis of the chloroplasts. The lysate was gently inverted several times every 15–20 min. Afterward, the lysate was cooled to room temperature. An equal volume of saturated phenol/chloroform/isopentyl alcohol (25:24:1) (Sinopharm Chemical Reagent Co., Ltd.) was added and gently mixed. The mixture was centrifuged at 12,000*g* for 10 min, and the supernatant was collected. Then, an equal volume of pre-cooled isopropanol was added and gently mixed. The mixture was allowed to stand at -20 °C for 10 min before being centrifuged at 12,000*g* for 20 min. The supernatant was discarded, and the residual alcohol was completely evaporated until there was no alcohol smell. Finally, the cpDNA was solubilized with sterile water.

An equal volume of 2% CTAB (cetyltrimethylammonium bromide) (Solarbio, China) [36] was added to the chloroplast suspensions obtained by the high-salt method and the NaOH low-salt method. The mixture was incubated at 55 °C for 1–2 h, and then the lysate was cooled to room temperature. Subsequently, an equal volume of saturated phenol/chloroform/isopentyl alcohol (25:24:1) was added and gently mixed. The mixture was centrifuged at 12,000*g* for 10 min, and the supernatant was collected. Then, an equal volume of pre-cooled isopropanol was added and gently mixed. The mixture was allowed to stand at -20 °C for 10 min before being centrifuged at 12,000*g* for 20 min. The supernatant was discarded, and the pellet was washed once with 70% ethanol and once with anhydrous ethanol. The residual alcohol was completely evaporated until no alcohol odor was present, and the cpDNA was dissolved with sterile water.

### DNA concentration, yield, and quality estimation

The DNA concentration was estimated by a UV spectrophotometer (Nanodrop ND-1000, Thermo, USA). Additionally, the extracted samples underwent electrophoresis on a 1.0% agarose gel (Tsingke, China) submerged in 1 × TAE (tris-acetate-EDTA) buffer (SCRC, China) at 180 V for 10 min. GelStain Blue (TransGen, China) was used for DNA staining, and visualization was performed using a UV gel imaging system (Syngene, UK). DNA quality was evaluated spectrophotometrically by measuring the optical density at A_230 nm_, A_260 nm,_ and A_280 nm_ with Nanodrop ND-1000 (Thermo, USA) equipment, as well as by electrophoresis. DNA was deemed to be of satisfactory quality if the A_260 nm_/A_230 nm_ ratio fell between 1.8 and 2.2 and the A_260 nm_/A_280 nm_ ratio ranged between 1.8 and 2.0 [37]. Furthermore, the presence of well-defined bands without smearing on the agarose gel was indicative of good DNA quality.

### qPCR analysis

Quantitative real-time PCR (qPCR) was conducted using THUNDERBIRD Next SYBR qPCR Mix (TOYOBO, Japan). The thermocycling conditions comprised denaturation at 95 °C for 1 min, followed by and 40 cycles of 95 °C for 5 s, 60 °C for 30 s, and 72 °C for 30 s. Samples were assayed in triplicate with 20 µL reactions, and the average Ct value was calculated.

The efficiency of the PCR reaction was assessed using standard curves generated from serial dilutions (1:10, 1:20, 1:40, and 1:80 dilutions) of the template. The efficiency (E) of each primer set was calculated for every DNA sample using the formula E = (10^− 1/slope^-1) × 100%. For each DNA sample, one nuclear gene (*β-actin*) and one chloroplast gene (*rbcL*) were amplified. Primer sets were designed for both the nuclear gene (*β-actin*) and chloroplast gene (*rbcL*), and preliminary experiments were conducted to select primer pairs yielding ideal dissolution curves and comparable amplification efficiencies (R^2^ values > 0.9) (Additional file 3). The formula (cpE^(cpCt)^)/(nE^(nCt)^) was employed to determine the relative number of chloroplasts compared to genomic copies. Primer sequences were designed using Primer3Plus software (EMBL Heidelberg) and are available in Additional file 1.

### Electronic supplementary material

Below is the link to the electronic supplementary material.

Supplementary Material 1

Supplementary Material 2

Supplementary Material 3

## Data Availability

The data and materials supporting the findings of this study are available from the corresponding author upon reasonable request.

## Abbreviations

cpDNA: chloroplast DNA
nDNA: nuclear DNA
PCR: polymerase chain reaction
qPCR: quantitative real-time PCR
MS: Murashige and Skoog
CTAB: cetyltrimethylammonium bromide
Tris-HCl: tris(hydroxymethyl)aminomethane hydrochloride
EDTA: ethylene diamine tetraacetic acid
PVP: polyvinylpyrrolidone
BSA: bovine serum albumin
DTT: dithiothreitol
SDS: sodium dodecyl sulfate
TAE: tris-acetate-EDTA
WHO: World Health Organization
TCM: traditional Chinese medicine
