# Supplementary material for: Extraction and analysis of high-quality chloroplast DNA with reduced nuclear DNA for medicinal plants
Source: BMC Biotechnol. 2024 Apr 18;24:20. doi: 10.1186/s12896-024-00843-8 (PMC11025248; doi:10.1186/s12896-024-00843-8)
Supplement: Supplementary file 1 — Supplementary Material 1 [file 12896_2024_843_MOESM1_ESM.pdf]

Supplementary Table 1

Supplementary Table 1 Oligonucleotides used for qPCR measurements

Gene	Primer name	Description	Sequence information (5′–3′)
rbcL	qrbcL-F	forward primer	GGGGATTCACTGCAAATACTACC
	qrbcL-R	reverse primer	CGTAAGGCTTTAGCTAGTACACG
β-actin	qPβ-actin-F	forward primer	AAGGATTCCTATGTGGGCGAC
	qPβ-actin-R	reverse primer	TTCTCCATGTCGTCCCAGTTG
