# Supplementary figures and images for: Extraction and analysis of high-quality chloroplast DNA with reduced nuclear DNA for medicinal plants
Source: BMC Biotechnol. 2024 Apr 18;24:20. doi: 10.1186/s12896-024-00843-8 (PMC11025248; doi:10.1186/s12896-024-00843-8)

### Supplementary Figure 1

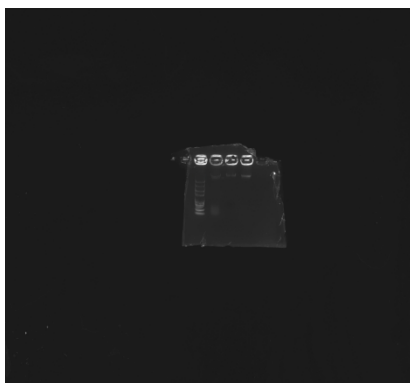

**Supplementary Figure 1:** unprocessed data related to Figure 3

Supplement: Supplementary file 2 — Supplementary Material 2 [file 12896_2024_843_MOESM2_ESM.pdf]
