# Supplementary material for: Extraction and analysis of high-quality chloroplast DNA with reduced nuclear DNA for medicinal plants
Source: BMC Biotechnol. 2024 Apr 18;24:20. doi: 10.1186/s12896-024-00843-8 (PMC11025248; doi:10.1186/s12896-024-00843-8)
Supplement: Supplementary file 3 — Supplementary Material 3 [file 12896_2024_843_MOESM3_ESM.docx]

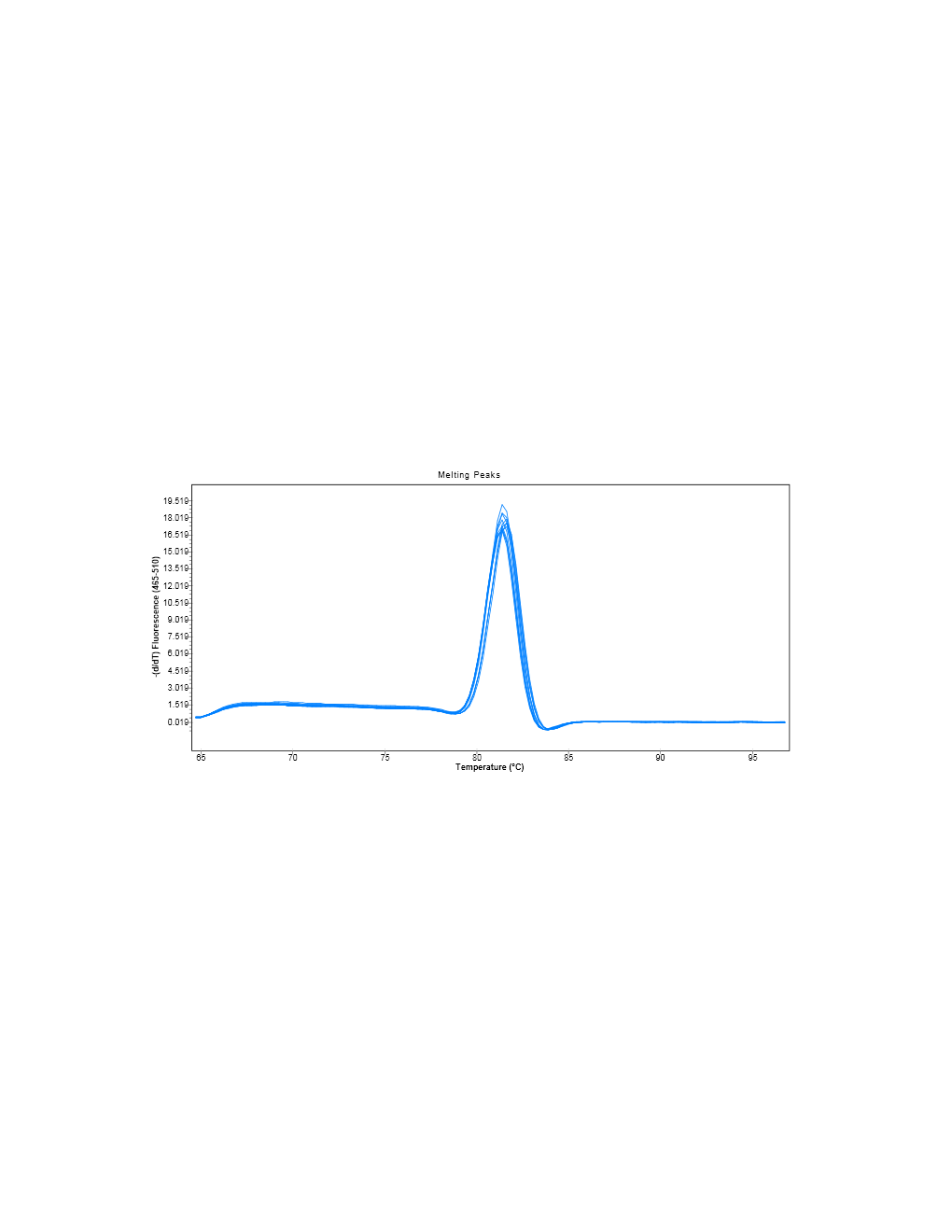


**Supplementary Figure 2** The melting curves of the primer pair for the *rbcL* candidate reference gene.

**Supplementary Table 2** The Ct values of the qPCR analysis of the primer pair for the *rbcL* candidate reference gene.

| Dilutions | Ct valus | Averaged Ct values |
| --- | --- | --- |
| 1:10 | 20.31 | 20.54666667 |
|  | 20.46 |  |
|  | 20.87 |  |
| 1:20 | 21.75 | 21.72333333 |
|  | 21.43 |  |
|  | 21.99 |  |
| 1:40 | 22.71 | 22.87666667 |
|  | 22.88 |  |
|  | 23.04 |  |
| 1:80 | 24.77 | 24.55333333 |
|  | 24.61 |  |
|  | 24.28 |  |

**
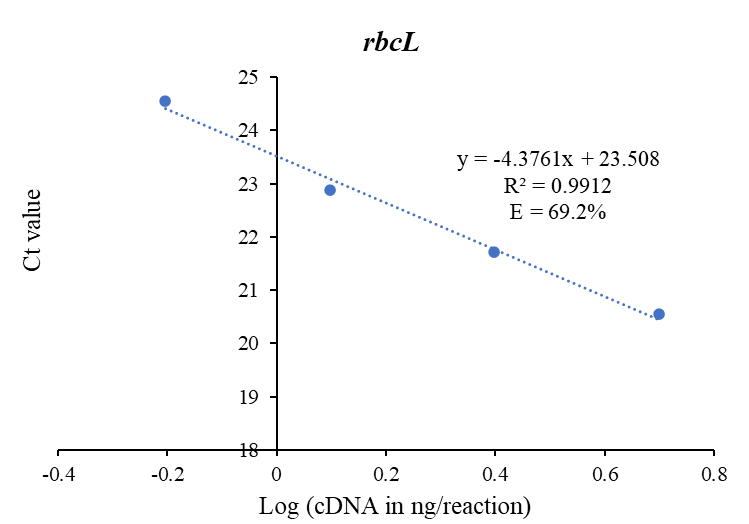
**

**S****upplementary Figure 3** The plot of the averaged Ct values obtained from three technical replicates plotted against the Log (cDNA in ng/reaction) for optimizing qPCR conditions for the primer pair of the *rbcL* candidate reference gene.

**
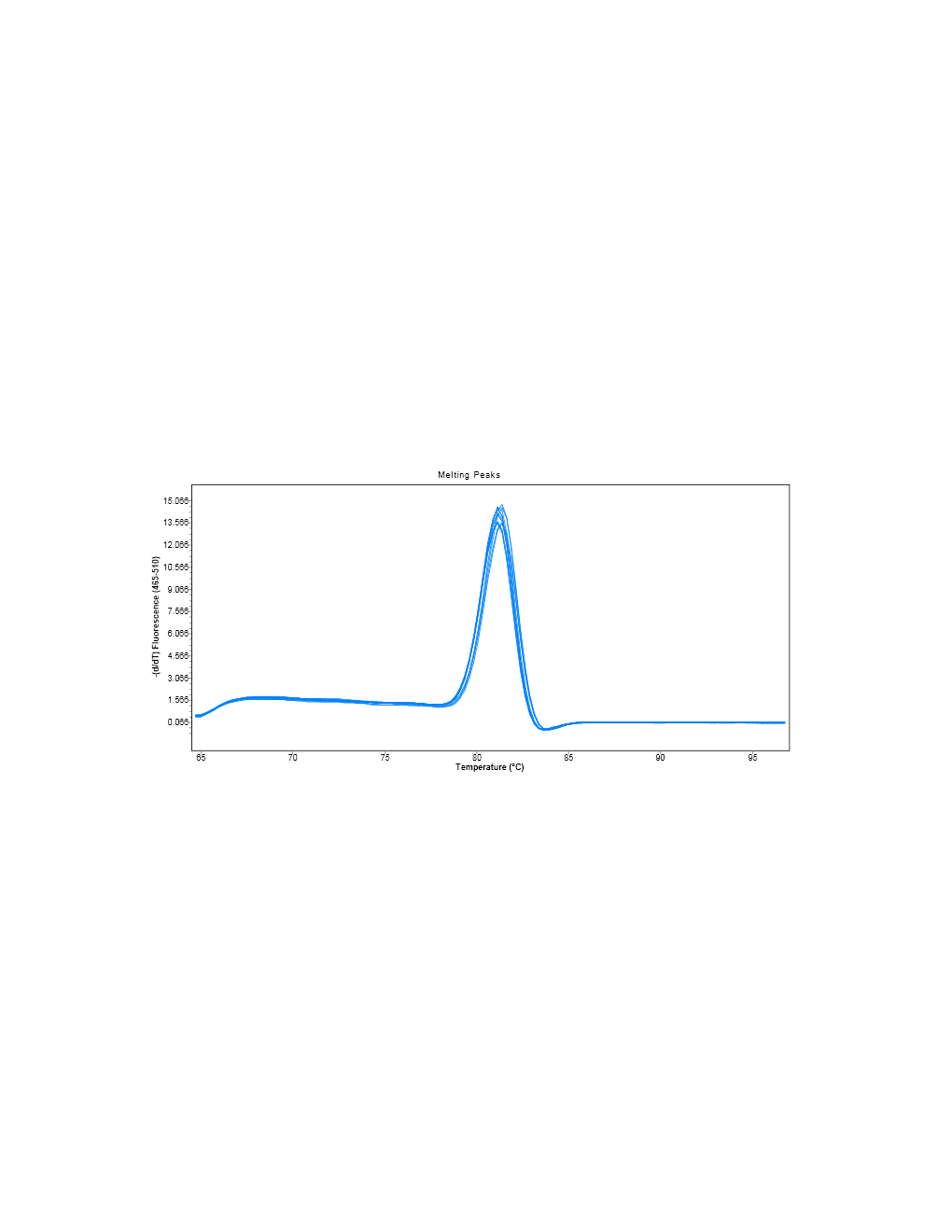
**

**Supplementary Figure 4** The melting curves of the primer pair for the *β-actin* candidate reference gene.

**Supplementary Table 3** The Ct values of the qPCR analysis of the primer pair for the *β-actin* candidate reference gene.

| Dilutions | Ct valus | Averaged Ct values |
| --- | --- | --- |
| 1:10 | 32.07 | 32.36 |
|  | 32.56 |  |
|  | 32.45 |  |
| 1:20 | 33.65 | 33.88 |
|  | 33.24 |  |
|  | 34.75 |  |
| 1:40 | 34.78 | 35.13 |
|  | 35.52 |  |
|  | 35.09 |  |
| 1:80 | 35.97 | 36.32 |
|  | 36.25 |  |
|  | 36.74 |  |

**
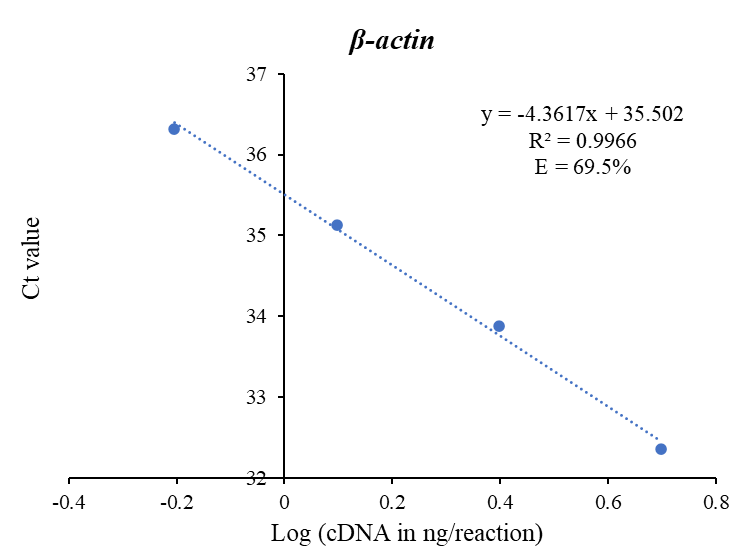
**

**Supplementary Figure 5** The plot of the averaged Ct values obtained from three technical replicates plotted against the Log (cDNA in ng/reaction) for optimizing qPCR conditions for the primer pair of the *β-actin* candidate reference gene.
